# Impact of sleep duration during pregnancy on the risk of gestational diabetes in the Japan environmental and Children’s study (JECS)

**DOI:** 10.1186/s12884-019-2632-9

**Published:** 2019-12-09

**Authors:** Mai Myoga, Mayumi Tsuji, Rie Tanaka, Eiji Shibata, David J. Askew, Yukiyo Aiko, Ayako Senju, Toshihiro Kawamoto, Toru Hachisuga, Shunsuke Araki, Koichi Kusuhara, Seiichi Morokuma, Masafumi Sanefuji, Japan Environment and Children’s Study, Hirohisa Saito, Reiko Kishi, Nobuo Yaegashi, Koichi Hashimoto, Chisato Mori, Shuichi Ito, Zentaro Yamagata, Hidekuni Inadera, Michihiro Kamijima, Takeo Nakayama, Hiroyasu Iso, Masayuki Shima, Yasuaki Hirooka, Narufumi Suganuma, Takahiko Katoh

**Affiliations:** 1 Department of Obstetrics and Gynecology, School of Medicine, University of Occupational and Environmental Health, 1-1 Iseigaoka, Yahatanishi-ku, Kitakyushu-city, Fukuoka, 807-8555 Japan; 2 Department of Environmental Health, School of Medicine, University of Occupational and Environmental Health, Fukuoka, Japan; 3 Japan Environment and Children’s Study, UOEH Subunit Center, University of Occupational and Environmental Health, Fukuoka, Japan; 4 Department of Pediatrics, School of Medicine, University of Occupational and Environmental Health, Fukuoka, Japan; 5 Research Center for Environmental and Developmental Medical Sciences, Kyushyu University, Fukuoka, Japan

**Keywords:** Gestational diabetes mellitus, Sleep duration in pregnancy

## Abstract

**Background:**

Gestational diabetes mellitus (GDM) has serious effects on both mother and child. Like Type 2 Diabetes Mellitus, it is increasing in prevalence world-wide. In addition to obesity, sleep duration has been named an important risk factor. Using a large cohort study, including data from 48,787 participants of the Japan Environment and Children’s Study (JECS), we examined the association between sleep duration and both random blood glucose levels and GDM rates during pregnancy.

**Methods:**

Random blood glucose levels were measured during pregnancy. GDM diagnosis was based on the results of 75 g oral glucose tolerance test. Additional anthropometric data was collected from questionnaires for statistical analysis.

**Results:**

Compared to mothers averaging 7 to < 10 h sleep (reference group), women receiving < 5 h or ≥ 10 h sleep exhibited significantly elevated random blood glucose levels. This was associated with an elevated risk for positive GDM screening (< 5 h sleep: OR 1.17 (0.96–1.44) *p* = 0.126; ≥10 h sleep: OR 1.13 (1.03–1.25) *p* = 0.006). Calculating the risk for GDM, women sleeping < 5 h or ≥ 10 h exhibited elevated risks of 1.31-fold and 1.21 respectively. However, this trend was not found to be significant.

**Conclusions:**

Sleep is a critical factor in glucose metabolism, with both abnormally long and short sleep duration increasing random blood glucose levels in pregnant women. Moreover, the risk for positive GDM screening increases significantly with elevated sleep, ≥10 h per night. These findings are promising because they support the idea that sleep duration is a modifiable risk factor, and can be focused upon to improve health and pregnancy outcome.

## Condensation

Abnormally long and short sleep duration increases random blood glucose levels, and sleep of ≥10 h per night increases the risk for positive GDM screening in pregnant women.

## Background

Gestational Diabetes Mellitus (GDM) is a serious health risk for both pregnant women and their offspring. GDM is prevalent worldwide, affecting ~ 13% of pregnancies. Rates of 20–25% are estimated for some countries of North Africa and the Middle East (United Arab Emirates) and the Western Pacific (Singapore and Thailand), whose medians are 12.9 and 11.7%, while rates are significantly lower in North America and many but not all of the European countries with estimated medians of 7.0 and 5.8% respectively [1]. In Japan, reported rates range from 1.8 to 13% [2, 3]. However, globally the Asian population accounts for some 60% of the diabetic population. In both Japan and China, rates of Type 2 Diabetes Mellitus (T2DM) has grown significantly over the past 20 years [4]. Similar to T2DM, the major risk factors for GDM include obesity and diet, hypertension, stress and inflammation. Likewise, GDM rates are also on the rise, increasing by more than 30% within the past 2 decades [5]. Due to the modifiable nature of several of its known risk factors, it is important to understand them and turn back its growing prevalence.

Sleep duration and quality are modifiable factors that have been linked to changes in glucose metabolism and T2DM [6]. Both epidemiology and animal models confirm that chronic sleep deficiency is a risk for metabolic-related diseases including obesity and impaired glucose tolerance, diabetes, and hypertension. As reviewed by Briancon-Marjollet A, et al., abnormal sleep patterns may lead to changes in hormone regulation, oxidative stress, inflammation, and adipogenesis-regulating signals [6]. All of these actions in turn can adversely affect glucose tolerance and metabolism.

Adequate sleep is commonly a challenge to maintain during pregnancy. Changes in sleep, both increases and decreases, are thought to be caused by hormonal changes, physical discomfort, and other pregnancy-related stresses [7, 8]. Indeed, a limited number of studies are now beginning to describe the predicted risk of changes in sleep pattern for developing GDM. As a pilot study, Qui C. et al. described an increased risk for GDM in women sleeping < 4 h vs. 9 h per night [9]. More recently, Flacco FL, et al. demonstrated significant ties between both short sleep duration and later sleep midpoints with GDM [10, 11]. In addition, an association between sleep-disorder breathing patterns and GDM was observed [11]. Meanwhile, Rawal S, et al. has identified a U-shaped relationship between sleep duration and GDM, defining both excessive as well as limited sleep amounts as risk factors for GDM. They also suggest that napping and pre-pregnancy obesity may be modifiers of this relationship [12].

Obesity is a risk factor on the rise in parallel with Diabetes rates in the Americas and European populations. However, while this risk factor is much lower in East Asian populations, it is observed that T2DM rates increase at a lower average body-mass index (BMI) in these populations [4]. It is therefore necessary to understand the role of additional modifiable factors in the battle against Diabetes. Using data collected by the large cohort Japan Environment and Children’s Study (JECS) we examined the effect of sleep duration on random blood glucose levels and GDM incidence during pregnancy within the Japanese population.

## Materials and methods

### Enrollment of participants

JECS is a nationwide, government funded birth cohort study begun in 2011 with the aim to elucidate the effects of environmental factors on the health of mother and child [13, 14]. 103,099 pregnancies were enrolled across the fifteen regional centers, which include representation of the diverse social, economic, and urban lifestyles of Japan during a 3-year period ending March 2014. The JECS protocol was approved nationally by the Institutional Review Board (IRB) in compliance with the Ethical Guidelines for Epidemiological Research, published by the Ministry of Education, Culture, Sports, Science and Technology, and the Ministry of Health, Labor and Welfare, and by the Ethics Committees at all participating institutions. Written informed consent was obtained from participants.

### Data collection

Two questionnaires per person were completed in the first (mean 16 weeks) and second to third trimesters (mean 27 weeks), addressing past pregnancy history, lifestyle, foods, and sleeping habits including sleep start and waking times. Specifically, sleep questions were administered in the second to third trimesters, and had the following phrasing, “What was the average time to get into bed and turn off the light over the last month?” and “What was your average waking time over the last month?”

Medical records were obtained, including maternal age, parity, height, pre-pregnancy body weight, body weight before delivery, blood glucose during pregnancy (random blood glucose, RBG, see below methods), gestational age at delivery, birth weight, and placental weight. Additional history, including information about Type1 or Type 2 DM or previous GDM diagnosis, and use of steroids during pregnancy were collected. Sleep duration, pre-pregnancy body-mass index (BMI) and gestational weight gain were calculated from raw data.

### Glucose tolerance and GDM screening

**Fig. 1:**
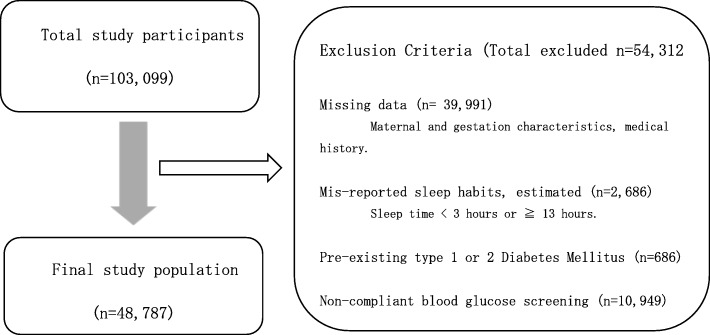
Study population inclusion characteristics

Glucose tolerance screening and testing for GDM was performed according to the protocols of the Obstetrics Society or Diabetes Society of Japan depending on the local institution using a 2-step protocol (Additional file 1: Figure S1). The first step was screening of random blood plasma glucose levels (RBG) during the first trimester. The second step was screening using either RBG or a fasting 50 g oral glucose challenge test (GCT) in the second trimester. An RBG score of ≧95 mg/dL or a 1 h 50 g GCT result of > 140 mg/dL were scored as a positive screening result. After a positive screening result, a 75 g oral glucose tolerance test (OGTT) was administered with GDM-positive cut-off values of fasting blood plasma glucose of ≤92 mg/dL, 1 h ≥ 180 mg/dL, or 2 h ≥153 mg/dL. In this study, for the purpose of consistency, we selected only subjects with RBG screening data (the majority 82%), to the exclusion of the fasting 50 g GCT-tested subjects (see Statistical analysis, Fig. 1).

### Statistical analysis

Participants were excluded based upon missing data (*n* = 39,991), non-compliant blood glucose screening results (10,949), existing type1 or type 2 diabetes (*n* = 686), and average sleep duration values less than 3 and over 13 h (*n* = 2686). Finally, 48,787 subjects were included in the analysis (see Fig. 1).

Sleep duration was categorized according to hours per night (< 5, 5 to < 7, 7 to < 10, and ≥ 10 h), with values estimated from sleep start and waking time survey responses. The 7 to < 10 h group was set as the reference. Because the random blood glucose level data did not have a normal distribution, the median (25th,75th) per sleeping duration were expressed, and the Mann-Whitney test based upon non-Gaussian distribution was used to compare sleep group blood glucose levels. Covariant factors included age, pre-pregnancy BMI, gestational weight gain [15], steroid use during pregnancy, and previous GDM. The adjusted relative risk ratios were calculated using a log-binomial regression model. Statistical analyses were completed using STATA version 11.1 (Texas, USA).

## Results

To begin our analysis, in addition to missing data, mis-reported sleep habits, and pre-existing T1 and T2DM, a large number of participants were excluded due to non-compliant blood glucose screening in order to remove variation associated with differences in screening and testing protocols (see Fig. 1) [2]. The resulting study group characteristics were similar to those of the original total participant population (Additional file 1: Table S1). The maternal characteristics of the study population (*n* = 48,787) are provided in Table 1. The values are similar to averages reported for the Japanese population recently, including values for pre-pregnancy BMI and gestational weight gain [16–18]. The study group contained 1000 GDM patients (2.05%). The rate of GDM is similar to rates previously observed in the Japanese population using the same diagnostic criteria [2, 3].

**Table 1:** Study population characteristics

	Total	GDM-negative	GDM
Number	48,787 (100%)	47,787 (97.95%)	1000 (2.05%)
Age (mean ± SD)	31.2 ± 4.99	31.1 ± 4.98	33.3 ± 5.00
Age (years)			
15–19		408 (0.8%)	3 (0.3%)
20–29		17,774 (37.2%)	241 (24.1%)
30–39		27,618 (57.8%)	651 (65.1%)
40-		1987 (4.2%)	105 (10.5%)
Pre-pregnancy BMI (mean)	21.2 ± 3.22	21.1 ± 3.15	23.7 ± 5.12
Pre-pregnancy BMI (kg/m2 ± SD)			
< 18.5		7750 (16.2%)	95 (9.5%)
18.5- < 25		35,373 (74.0%)	590 (59.0%)
≧25		4664 (9.8%)	315 (31.5%)
Parity (time)	0.87 ± 0.89	0.87 ± 0.89	0.92 ± 0.96
gestational weight gain (kg)	10.3 ± 3.93	10.4 ± 3.89	7.89 ± 5.16
Gestational age at delivery (weeks)	38.8 ± 1.47	38.8 ± 1.47	38.5 ± 1.62
Birth weight (g)	3027 ± 407	3027 ± 406	3028 ± 444
Placenta weight (g)	563 ± 119	563 ± 119	574 ± 121

Participant values are presented with percent of group (%). Average values are presented ±standard deviation

Estimated daily sleep duration ranged between 3 and 13 h per day. The median random blood glucose (RBG) levels for each sleep category are presented in Table 2. Pregnant mothers in the reference group of 7 to < 10 h sleep per night (84 mg/dL (77, 94)), and the group receiving 5 to < 7 h of sleep (median 84 mg/dL (77, 94)) had the lowest median RBG levels. Women receiving < 5 h or ≥ 10 h of sleep exhibited significantly elevated glucose levels compared to the 7 to < 10 h sleep reference group using the Mann-Whitney test based upon non-Gaussian distribution (Table 2). This observation describes a U-shaped relationship, with sleep times at both the short and long end of the scale associated with higher RBG levels.

**Table 2:** Mean RBG associated with sleep duration

Sleep time (hr)	N	Median random blood glucose (mg/dL)	P ^*a*^
< 5	468	85 (78, 96)	0.042*
5 to < 7	8897	84 (77, 94)	0.109
7 to < 10	36,836	84 (77, 94)	Ref.
≥10	2586	85 (77, 96)	0.033*

*a*. *P* value was determined using the Mann-Whitney test base upon non-Gaussian distribution of data

* Significant *p* ≤ 0.05

Comparing the positive-screening rates between the four sleep duration categories (Table 3), significant increases were observed for women sleeping ≧10 h (OR 1.13 (95%CI = 1.03–1.25), *p* = 0.006), compared to the reference group of 7 to < 10 h sleep per night. An elevated risk was also observed in the < 5 h sleep group (OR 1.17 (0.96–1.44)), however the statistical significance of this association fell short (*p* = 0.126).

**Table 3:** Risk for positive GDM screening associated with sleep duration

	GDM Pre-screening result ^*a*^				
	Negative (*n* = 36,908)	Positive (*n* = 11,879)	OR ^*b*^	95%CI	P
Sleep time (hours)					
5 <	336	132	1.17	0.96–1.44	0.126
5 to < 7	6807	2090	0.95	0.90–1.00	0.047 *
7 to < 10	27,860	8976	Ref.	Ref.	Ref.
≥ 10	1905	681	1.13	1.03–1.25	0.006 *

*a*. GDM Pre-screening proceeded a diagnostic 75 g OGTT test. Positive result was a ≧95 mg/dL resting glucose score

*b*. Adjusted relative risk (95% CI) for positive GDM screening associated with sleep duration, confounding factors included age, pre-pregnancy BMI, gestational weight gain [12], steroid use during pregnancy, and previous GDM. The adjusted relative risk ratios were calculated regression mode

* Significant p ≤ 0.05

Finally, the risk for GDM diagnosis relative to sleep duration was determined using a log-binomial regression model. Women sleeping < 5 h were found to have 1.30-fold risk for GDM. Similarly, women sleeping ≧10 h had a 1.21-fold risk for developing GDM compared to the reference group (Table 4). However, these observations failed to meet the significant value cut-off. Therefore, the effects of elevated or limited sleep observed directly on blood glucose levels did not translate into significant increases in GDM incidence in this Japanese population.

**Table 4:** Risk for GDM associated with sleep duration

	GDM				
	no (*n* = 47,787)	yes (*n* = 1000)	OR ^*a*^	95%CI	P
Sleep time (hours)					
< 5	454	14	1.31	0.74–2.30	0.353
5 to < 7	8703	194	1.03	0.87–1.22	0.742
7 to < 10	36,096	740	Ref.	Ref.	Ref.
≥ 10	2534	52	1.21	0.90–1.63	0.207

*a.* Adjusted relative risk (95% CI) for GDM associated with sleep duration. Confounding factors included age, pre-pregnancy BMI, gestational weight gain [12], steroid use during pregnancy and previous GDM

* Significant p ≤ 0.05

## Discussion

This study utilized a large cohort study representing the population of Japan to focus on defining the effects of sleep duration on glucose metabolism and the risk of GDM [13]. Participant data was sorted based on estimated sleep duration into 4 categories. As previously observed, this study’s median random blood glucose levels increased with both decreased and increased sleep duration, following a U-shaped association with respect to sleep. Using the Japanese national guidelines in their two-step screening protocol, a significant relationship between sleep duration and positive screening results was also identified. Excessive sleep, ≥10 h was associated with an increased risk (OR = 1.13 (95%CI = 1.03–1.25), *p* = 0.006), compared to the reference group. An elevated risk in the limited, < 5 h sleep group, although non-significant, was also observed (OR 1.17 (0.96–1.44), *p* = 0.126). The significant association measured here between excessive or limited sleep and blood glucose metabolism failed to translate into the increased GDM diagnosis rates that are predicted. Indeed, only modest increases could be determined for the < 5 h (OR = 1.31 (0.74–2.30), *p* = 0.353) and ≥ 10 h (OR = 1.21 (0.90–1.63), *p* = 0.207) sleep duration groups.

Based upon similar studies, it was surprising that this large cohort data set did not reveal a significant relationship between sleep and GDM rates. By an alternative analysis, using eight, 1-h sleep duration categories, the same trend was revealed as that using four categories (see Additional file 1: Table S2). Moderate sleep groups of 6, 7 (reference), 8, and 9 h exhibited lower GDM-risk rates (OR = 0.93, (ref.), 0.88, and 0.9 respectively). Meanwhile, excessive sleep, ≥10 h (OR = 1.13), or minimal sleep, 3 h (OR = 4.05 (1.68–9.80), *p* = 0.002), were both associated with elevated risk of GDM diagnosis. In the case of minimal sleep, this elevated risk was found to be significant.

These findings support initial reports in the literature which describe how both sleep quantity and quality during pregnancy are important GDM risk factors [9, 10, 12, 19–22]. Together we present strong support for the idea that both restricted as well as excessive sleep is a risk for the loss of proper glucose metabolism and control during pregnancy.

Obesity and the rising rate of diabetes, including Type 2 diabetes mellitus (T2DM) and GDM is a global medical concern. Importantly, there is evidence for risk factors that are associated with different ethnic groups and races [7]. Two studies examining sleep duration and GDM, conducted with ethnically mixed populations in America, found that sleep duration was significantly related to ethnicity and race [11, 12]. This study is unique because it is focused on the East Asian population of Japan, a population which, despite low rates of obesity and weight gain during pregnancy, has a growing rate of T2DM and GDM [23]. The screening protocol of the Japanese Obstetrics Society is the same in most aspects to that of the International Association of the Diabetes and Pregnancy Study Groups (IADPSG), and so our results are applicable to populations worldwide.

While the size of this study allowed us to draw significant conclusions about the relationship between sleep quantity and blood glucose metabolism, this study is preliminary in regards to sleep and GDM incidence in Japan. Future questionnaires should include a validated sleep survey to better quantify sleep duration. In addition, data on related, confounding factors not accounted for in this study, such as sleep quality, a primary risk factor of GDM which acts through changing glucose metabolism should be collected [10, 12].

The variability that is inherent in a large, nationwide cohort study was also likely to adversely affect our outcome. Designed to represent the diverse living conditions and areas of Japan, data collection was dependent on a large number of institutions, ranging from university research hospitals to small obstetrics clinics [13]. Even in the case of applying the same protocol there is often significant variation in measurements reported between institutions [24]. Finally, at the time of this study the standard protocol for screening and final determination of GDM were in transition in Japan, based upon the recommendation of the Japan Obstetrics Society [25]. In the process of this analysis a large number of participants needed to be excluded from the final study group to insure a single universal GDM protocol was used. Indeed, the universalization of protocols and diagnostic criteria has been a challenge in the field of Diabetes and GDM research.

## Conclusions

This investigation aides the current effort to identify and define modifiable factors associated with the growing global threat of Diabetes. Our findings of a U-shaped association between sleep duration and glucose metabolism, support other current reports. In addition, this report importantly adds unique data from the East Asian population of Japan to help understand racial differences, as well as to address the growing proportion of Diabetes cases associated with this population relative to the entire world.

## Supplementary information

**Additional file 1 Fig. S1.** Glucose Tolerance and Gestational Diabetes Screening schedule. **Table S1.** Comparison of the study group and original total population characteristics. **Table S2.** Risk for GDM associated with sleep duration.

**Additional file 2.** Supplementary Ethics Committee titles of all participating institutions.

## Data Availability

The JECS data are not publicly available due to ethical restriction and the legal framework of Japan. All inquiries about access to the data should be sent to the JECS Program Office, National Institute for Environmental Studies (jecs-en@nies.go.jp).

## Abbreviations

BMI: Body-mass index
GCT: Glucose challenge test
GDM: Gestational Diabetes Mellitus
IADPSG: International Association of the Diabetes and Pregnancy Study Groups
IEC: Independent Ethics Committee
IRB: Institutional Review Board
JECS: Japan Environment and Children’s Study
JSOG: Japan Society of Obstetrics and Gynecology
OGTT: Oral glucose tolerance test
RBG: Random blood glucose
T1 or T2DM: Type 1 or Type 2 Diabetes Mellitus
