# Supplementary material for: Impact of sleep duration during pregnancy on the risk of gestational diabetes in the Japan environmental and Children’s study (JECS)
Source: BMC Pregnancy Childbirth. 2019 Dec 9;19:483. doi: 10.1186/s12884-019-2632-9 (PMC6902452; doi:10.1186/s12884-019-2632-9)
Supplement: Supplementary file 1 — Additional file 1 Fig. S1. Glucose Tolerance and Gestational Diabetes Screening schedule. Table S1. Comparison of the study group and original total population characteristics. Table S2. Risk for GDM associated with sleep duration. [file 12884_2019_2632_MOESM1_ESM.docx]

***Supplementary Figure 1.*** Glucose Tolerance and Gestational Diabetes Screening schedule.

Second Step, 2^nd^ Trimester screening:

RBG or 50g Oral glucose tolerance test

(OGTT cut off: 1hr >140mg/dL)

First Step, 1^st^ Trimester screening:

Random blood plasma glucose (RBG)

(cut off: >=95mg/dL)

Positive

Negative

Negative

Gestational Diabetes Mellitus screening:

(Fasting BG screen and/or) 75g Oral glucose tolerance test

(GDM cut off: Fasting BG <=92mg/dL, OGTT 1hr >=180mg/dL, or 2hr >=153mg/dL)

***Supplementary Table 1*.** Comparison of the study group and original total population characteristics

|  | n=48,787 | n=103,099 |
| --- | --- | --- |
| Age (years) | 31.2±4.99 | 31.0±5.04 |
| Pre-pregnancy BMI (kg/m^2^) | 21.2±3.22 | 21.1±3.74 |
| Parity (time) | 0.87±0.89 | 0.86±0.90 |
| gestational weight gain (kg) | 10.3±3.93 | 7.52±13.8 |
| Gestational age at delivery (weeks) | 38.8±1.47 | 38.4±3.27 |
| Birth weight (g) | 3027±407 | 2996±484 |
| Placenta weight (g) | 563±119 | 561±125 |
| Frequency of GDM (%) | 2.05 | 2.69 |

***Supplementary Table 2.*** Risk for GDM associated with sleep duration

|  | GDM | | |  |  |  |
| --- | --- | --- | --- | --- | --- | --- |
|  | no  (n=47,787) | | yes  (n=1,000) | OR *^a^* | 95%CI | p |
| Sleep time (hours) | |  |  |  |  |  |
| 3 to <4 | | 68 | 6 | 4.05 | 1.68-9.80 | 0.002 * |
| 4 to <5 | | 386 | 8 | 0.78 | 0.37-1.63 | 0.506 |
| 5 to <6 | | 1,810 | 46 | 1.10 | 0.79-1.52 | 0.577 |
| 6 to <7 | | 6,893 | 148 | 0.93 | 0.75-1.14 | 0.465 |
| 7 to <8 | | 14,981 | 325 | Ref | Ref. | Ref |
| 8 to <9 | | 14,067 | 277 | 0.88 | 0.75-1.05 | 0.149 |
| 9 to <10 | | 7,048 | 138 | 0.90 | 0.73-1.11 | 0.340 |
| ≥10 | | 2,534 | 52 | 1.13 | 0.83-1.54 | 0.433 |

*a*. The adjusted relative risk ratios were calculated regression model. Confounding factors included age, pre-pregnancy BMI, gestational weight gain [12], steroid use during pregnancy and previous GDM.

* Significant p≤ 0.05.
