# Supplementary material for: Impact of sleep duration during pregnancy on the risk of gestational diabetes in the Japan environmental and Children’s study (JECS)
Source: BMC Pregnancy Childbirth. 2019 Dec 9;19:483. doi: 10.1186/s12884-019-2632-9 (PMC6902452; doi:10.1186/s12884-019-2632-9)
Supplement: Supplementary file 2 — Additional file 2. Supplementary Ethics Committee titles of all participating institutions. [file 12884_2019_2632_MOESM2_ESM.docx]

Supplementary Ethics Committee titles of all participating institutions

1) Independent Ethics Committee (IEC) of the National Center for Child Health and Development, Hokkaido University

2) Institutional Review Board (IRB) of Sapporo Medical University

3) IEC of the Asahikawa Medical College

4) IEC of the Japanese Red Cross Hokkaido College of Nursing

5) IEC of Tohoku University

6) IEC of Fukushima Medical University

7) IRB of Chiba University

8) IEC of Yokohama City University

9) IEC of the University of Yamanashi

10) IEC of Shinshu University
11) The Ethics Committee of Toyama University

12) IRB of Nagoya City University
13) IEC of Kyoto University

14) The Doshisha University Research Ethics Review Committee Regarding Human Subject Research
15) IEC of Osaka University
16) IEC of Osaka Medical Center and Research Institute for Maternal and Child Health

17) IEC of Hyogo College of Medicine

18) IRB of Tottori University
19) The Research Ethics Committee of Kochi University

20) IRB of The University of Occupational and Environmental University

-origination of this study)

21) IEC of Kyushu University

22) IEC of Kumamoto University
23) IEC of the University of Miyazaki

24) IEC of the University of the Ryukyus
